# From Critical Thinking to Well-Being: The Metacognitive Pathway in a Graphical-Causal Model

**DOI:** 10.3390/jintelligence14090193

**Published:** 2026-09-01

**Authors:** Carlos Saiz, Miguel H. Guamanga, Silvia F. Rivas, Cristiano M. A. Gomes

**Affiliations:** 1 Department of Basic Psychology, Psychobiology and Methodology of Behavioral Sciences, University of Salamanca, 37005 Salamanca, Spain; silviaferivas@usal.es; 2 Faculty of Human Sciences, Universidad Icesi, Cali 760000, Colombia; miguel.guamanga@u.icesi.edu.co; 3 Laboratory for Cognitive Architecture Mapping (LAICO), Federal University of Minas Gerais, Belo Horizonte 31270-901, Brazil; cristianogomes@ufmg.br

**Keywords:** critical thinking, metacognition, psychological well-being, academic performance, directed acyclic graphs

## Abstract

This study reanalyzes data from a previous investigation on critical thinking, metacognition, empathy, and psychological well-being by incorporating academic performance into a graphical-causal model. The aim was to compare two explanatory structures: a performance-mediated model, in which metacognition relates to psychological well-being through academic performance, and a direct metacognitive model, in which metacognition maintains a direct relation with well-being. The sample included 155 undergraduate psychology students. Critical thinking, metacognition, empathy, academic performance, and psychological well-being were modeled using directed acyclic graphs, conditional independence tests, Gaussian Bayesian networks, and bootstrap-based arc stability analysis. The initial performance-mediated DAG was not supported because the implied independence between metacognition and well-being conditional on performance was violated. The refined model, which included a direct metacognition–well-being path, was more compatible with the data. Metacognition showed the strongest estimated path to psychological well-being, whereas academic performance did not operate as the main mediating mechanism. Bootstrap results supported the stability of the metacognition–well-being adjacency. Overall, the findings suggest that critical thinking relates to psychological well-being mainly through metacognitive processes, under the assumptions of the specified DAG.

## 1. Introduction

In recent years, higher education has had to adapt to increasingly rapid technological change: first, the spread of smartphones and, subsequently, the mass expansion and use of social media; and, more recently, the emergence and development of generative artificial intelligence, especially since the public release of ChatGPTv1.0 in late 2022 (OpenAI, 2022). These changes have brought a central question for university education back to the foreground: what to teach, why to teach it, and how to teach in a context in which the production, reorganization, and circulation of information are increasingly supported by automated systems whose interests remain opaque (Bender & Hanna, 2025) and whose impact on education remains under discussion (Bai et al., 2023; Qian, 2025; Raitskaya & Tikhonova, 2025).

Thus, in the face of automated systems capable of performing routine tasks, the current pedagogical challenge is to educate individuals who can critically evaluate information, make deliberate decisions, and act reasonably in the deeply uncertain and ambiguous environments of contemporary society. In other words, both before and after the age of artificial intelligence, the fundamental aim of education has been—and will continue to be—learning to think. In this regard, Resnick’s (1987) thesis remains notably current: education should be oriented toward the development of higher-order thinking, which is characterized as complex, non-algorithmic, and capable of managing uncertainty to construct meaning.

From this perspective, critical thinking instruction is especially urgent today. If critical thinking is understood as one of the strongest tools for explaining reality and solving problems (Davies & Barnett, 2015; Dwyer, 2023; Ennis, 2011; Halpern & Dunn, 2023; Hamby, 2015; Saiz, 2020; Sternberg, 2021; Sternberg et al., 2024), its importance does not diminish in the age of artificial intelligence; rather, it increases. The availability of automated responses requires a greater capacity to evaluate the quality of explanations, distinguish among reasons, causes, and motivations, detect biases, weigh alternatives, and anticipate consequences. Therefore, the question is not whether artificial intelligence replaces critical thinking. The educational challenge is how to strengthen it in an environment where students interact with artificial systems without delegating to them understanding, decision-making, or responsibility for their actions.

Addressing education beyond cognitive components is a complex and debatable issue; however, in the present case, it is essential. As several contemporary approaches have noted, critical thinking as an educational competence is not limited to exclusively cognitive components (Dwyer, 2017, 2023; Facione, 1990; Halpern & Dunn, 2023; Saiz, 2020; Sternberg, 2021). In the 1980s, Ennis (1987, 1996) emphasized that the development of critical thinking must take into account both cognitive abilities and dispositions, or non-cognitive components, such as open-mindedness in seriously considering other points of view, persistence in being well informed, and the willingness to change one’s mind when evidence and reasons are sufficient. The distinction between these two components is especially important in the present context because decision making depends not only on the inferential quality of a response—a task that may be partly addressed through the learning of logic—but also on value orientation, responsibility, sensitivity to consequences, and willingness to revise one’s own judgment. Artificial intelligence can facilitate the search, synthesis, and generation of information. Still, it cannot replace the normative and self-regulatory dimension involved when a person decides what to accept, what to reject, and how to act in response to a problem (Baron, 2024; Halpern & Dunn, 2021; Saiz & Rivas, 2023).

Against this background, the present study examines whether the relation between critical thinking and psychological well-being is better understood through academic performance or through metacognitive processes. To address this question, we reanalyze data from a previous study by incorporating academic performance into a graphical causal model and by comparing two theoretically plausible structures: a performance-mediated pathway and a direct metacognitive pathway. The significance of this approach lies in moving beyond a purely associative interpretation of previous SEM findings and evaluating whether the structural assumptions of each model generate conditional independence implications compatible with the observed data. In this way, the study contributes to current debates on critical thinking in higher education by clarifying whether grades or metacognitive self-regulation provide the more coherent explanation of the link between critical thinking and psychological well-being.

## 2. Literature Review and Theoretical Background

This section organizes the theoretical and empirical background that supports the graphical causal model evaluated in the present study. The review first situates critical thinking within the transition from argumentation-centered approaches to explanation-, decision-, and problem-solving-oriented approaches. It then examines metacognition as a monitoring and self-regulatory process involved in planning, controlling, and evaluating one’s own reasoning and learning. These processes are especially relevant in higher education, where students must not only achieve academic outcomes but also regulate their learning, interpret their experiences, and sustain psychological well-being. The section then reviews the roles of academic performance, empathy, and psychological well-being as educational and socioemotional outcomes, before presenting the rationale for extending previous SEM evidence through graphical causal modeling, directed acyclic graphs, and conditional independence testing.

### 2.1. Critical Thinking: From Argumentation to Explanation

Critical thinking has traditionally been associated with argumentation, informal logic, the evaluation of reasons, and the justification of conclusions. From this perspective, Toulmin’s (2003) model has decisively shaped the development of the field by establishing argumentative structure—data, warrant, backing, modal qualifier, rebuttal, and claim—as a preferred framework for the analysis and evaluation of arguments. This tradition has played an important role in consolidating critical thinking as an object of education and research (Hitchcock, 2017; Kuhn, 1999; van Gelder, 2015). However, it is insufficient for understanding the role of critical thinking in contexts in which it is not enough to justify a conclusion, but is also necessary to explain, decide, and solve everyday problems in professional or personal settings (Baron, 2024; Dumitru & Halpern, 2023; Saiz, 2020; Saiz & Rivas, 2023).

In argumentation, the central concern is justification, that is, supporting a claim through reasons. Explanation, by contrast, focuses on causes: the effect is the fact, phenomenon, or problem to be understood, but the decisive issue is to determine why it occurs. From this perspective, argumentation should serve explanation, since it helps organize and evaluate the reasons that can shorten the path of inquiry toward the causes of the problem (Guamanga et al., 2023; Saiz, 2020). Therefore, the development of critical thinking should be limited neither to argumentative analysis nor to understanding a situation; it should move toward causal explanation, decision-making, and problem-solving (Saiz & Rivas, 2012).

This expansion makes it possible to distinguish two approaches to critical thinking: one based on argumentation theory and another centered on action theory (Saiz & Rivas, 2012). The former is mainly oriented toward the presentation and justification of a claim; the latter also requires transforming reality through decisions and actions. In the first case, pre-decisional components predominate; in the second, critical thinking integrates both pre-decisional and post-decisional processes. Explaining reality provides the causal knowledge needed to make more informed decisions and to implement effective problem-solving strategies. Accordingly, the formative sequence on which this study is based can be expressed as follows: explain, decide, solve, and generate change.

However, this relation cannot be reduced to a purely cognitive process. As thinking moves toward decision and action, dispositions, values, convictions, attitudes, responsibility for consequences, and perception of context come into play. Non-cognitive components are difficult to observe and modify, yet they are indispensable for understanding why a person decides to act—or not to act—in accordance with their reasoning. At this point, counterfactual thinking offers an important conceptual bridge: it allows individuals to imagine causally plausible alternative scenarios, anticipate consequences before making a decision, and evaluate whether a course of action is acceptable in light of specific goals, values, and responsibilities (Pearl & Mackenzie, 2018). Therefore, an applied understanding of critical thinking should integrate causal explanations, counterfactual simulations, and dispositions oriented toward responsible action (Baron, 2024; Ennis, 2011; Saiz & Rivas, 2023).

**Figure 1:**
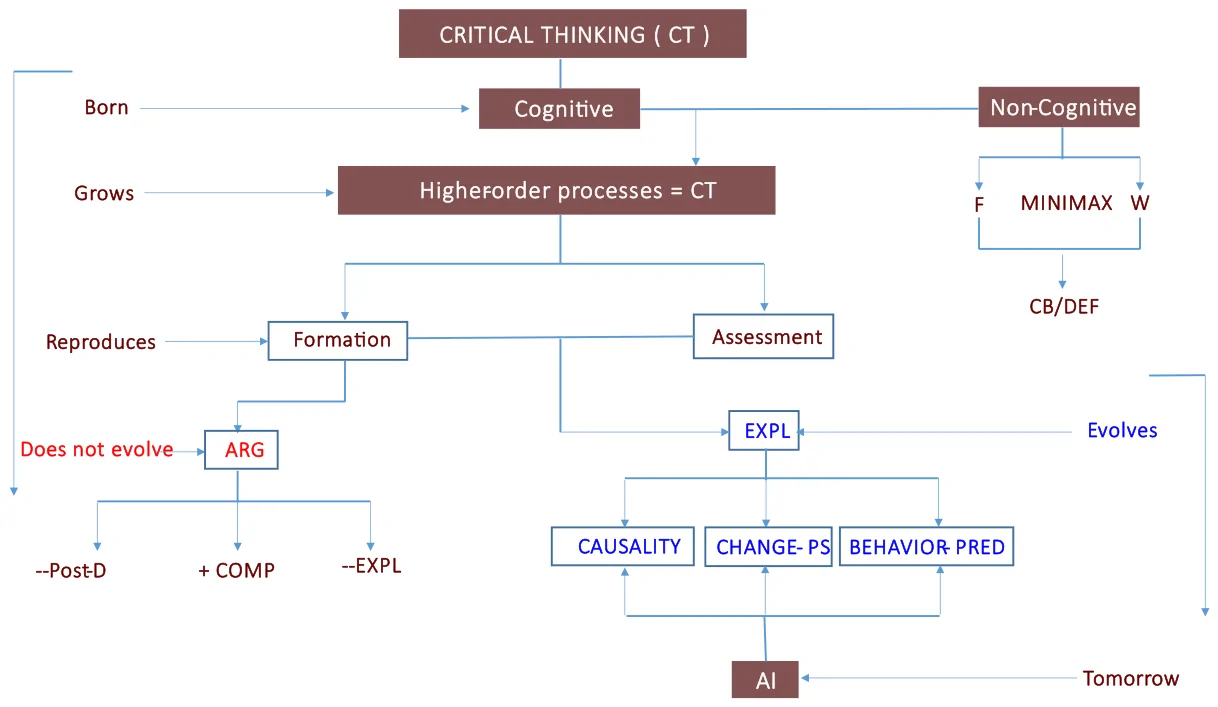
Conceptual evolution of critical thinking. *Note.* MINIMAX: The Law of MINImal Effort and MAXimum Reward. F: Feeling. W: Willingness. CB: Confirmation Bias. DEF: DEFiciencies. ARG: ARGumentation. EXP: EXPLanation. Post-D: Post-Decision. COMP: COMPrehension. PS: Problem Solving. PRED: PREDition.

Figure 1 summarizes this conceptual development. The aim is not to exclude argumentation from critical thinking, but to place it within a broader system. Argumentation helps evaluate the coherence of reasoning, whereas causal explanation makes it possible to understand which conditions give rise to a problem and which measures might modify it.

In Figure 1, we depict the evolution of the CT along two somewhat different paths: one leading to stagnation (no evolution) and the other allowing for evolution. But let’s go back to the origins. We can trace its origins to the work of Toulmin (1958, 2003), in which he proposes a model based essentially on argumentation—or, in other words, on informal logic. Therefore, here its nature is exclusively cognitive. Toulmin’s model inspired most CT models until about two decades ago (see Baron, 2024). Taking a different approach, such as that of Robert Ennis in the 1990s, this author incorporated non-cognitive components into the CT (Ennis, 1996). Thanks to this contribution, the limitations, biases, and deficiencies (DEF) of our cognitive system began to be considered. The principle of minimum effort and maximum gratification (MINIMAX) is of enormous adaptive importance. This principle optimizes positive emotions and minimizes effort to achieve pleasurable feelings (F) and cognitive economy, driven by will (W) or motivation. We all know that thinking is tiring, so we reduce our effort and look for shortcuts to achieve our goals. Of course, the greatest enemy of our processing system is confirmation bias (CB), a phenomenon widely studied in the literature and, therefore, familiar to most scholars of cognitive science.

Continuing with our analysis, and with the help of Figure 1, the growth of CT is primarily due to a better understanding of higher-order processes—everything that cognitive research has contributed since the 1980s. However, efforts have always been made to develop or improve this knowledge; this applied aspect of CT is a constant. For this reason, training initiatives have generally been in place. However, it must be noted that they have been accompanied by very little evaluation—a particularly significant gap in approaches where argumentation (ARG) plays the leading role (Saiz & Rivas, 2023). Consequently, the growth and expansion of the field of CT have been limited along this path due to the lack of measurement of the effects of these interventions. It is difficult to know whether anything improves if we do not evaluate the training. Not only do we face a limitation in evaluating its achievements along this path, but there is also another limitation regarding its priorities. By placing argumentation at the center of CR, other higher-order processes are minimized; in some cases, they are not considered components of CR. The Center for Research in Reasoning, Argumentation & Rhetoric (CRRAR: www.uwindsor.ca/crrar; accessed on 15 February 2016) is a good example of this approach. From this perspective, little importance is given to post-decision (—Post-D) processes—that is, to problem-solving and decision-making strategies. Furthermore, understanding (+COMP) is prioritized, while explanation (-—EXPL) is downplayed. Epistemologically speaking, causality is relegated to a secondary role (for a systematization of developments in CR training, see Saiz, 2024).

We agree that the development of CT, through argumentation and all its determining factors, should seek the truth. In fact, at its core, a theory or model of argumentation, formally speaking, does not differ from explanation in terms of inferential mechanisms. We also consider this formal research and development to be important and illuminating. However, this ideal world of the formal does not function like the real world of causality. In reality, we need to know what causes what, because without a precise explanation of the realities that influence or affect us, we will never be able to avoid the negative consequences or problems they might produce. What happens in our world, combined with sound reflection, is what provides us with the most important knowledge as human beings. Facts are what determine the correctness of our inferences and allow us to grasp—though not necessarily fully comprehend—the causes of events. Ultimately, the difference between these two paths in the development of critical thinking stems from their objectives: the search for truth and problem-solving. These objectives obviously determine why one perspective does not evolve, while the other does. The ideal world of reflection requires only an effort to achieve the best representation of reality; the real world of causality, however, also requires modifying that reality. As we mentioned earlier, we could describe these two ways of understanding the CT as a theory of argumentation in one case and a theory of action in the other. In the former, we need only pre-decisional (purely representational) components, and in the latter, both pre- and post-decisional components (see the description below). Limiting ourselves to the representational aspect alone—or, at most, considering the post-decisional aspect merely as complementary—does little to help us address the dramatic changes currently taking place in our world. Theoretical reflection alone does not enable us to understand, explain, or modify reality; we need other actors who take action, who implement or bring those accurate representations of reality to life. Therefore, for us, critical thinking means arriving at the best explanation for a fact, phenomenon, or problem to understand and resolve it effectively (Saiz, 2020, p. 27).

This distinction is particularly important for the present study because it allows critical thinking to be connected with metacognitive monitoring and regulation processes, as well as with observable pedagogical and psychological outcomes such as academic performance, empathy, and psychological well-being.

### 2.2. Metacognition as a Process of Monitoring and Regulation in Critical Thinking

Metacognition is a central mechanism for understanding how critical thinking relates to other educational and socioemotional outcomes. In general terms, it involves awareness, monitoring, regulation, and evaluation of one’s own cognitive processes (Flavell, 1979; Schraw & Dennison, 1994; Schraw & Moshman, 1995). In the context of critical thinking, metacognition allows individuals to plan courses of action, monitor the quality of their inferences, detect errors, adjust strategies, and evaluate the effectiveness of their decisions (Halpern, 1998; Kuhn & Dean, 2004).

The approach adopted in the present study is grounded in a basic pedagogical idea: it does not seem possible to develop a complex skill without metacognition. Any improvement in a competence requires at least two prior conditions: some degree of awareness of one’s own mental processes and adequate planning and self-regulation of the sequences of actions oriented toward problem solving (Guamanga et al., 2025; Rivas et al., 2022; Ryan & Deci, 2000; Saiz, 2024). In this sense, metacognition can be understood as an “awareness” of what occurs in one’s own thinking and, at the same time, as the strategic organization of those thinking processes.

The relation between critical thinking and metacognition can be formulated in more than one direction. On the one hand, metacognition appears to be necessary for critical reasoning because it allows individuals to monitor and revise their own thinking. On the other hand, from a formative and synchronic perspective, critical thinking may foster metacognitive development, insofar as tasks involving explanation, decision making, and problem solving require students to examine their inferences, evaluate evidence, compare alternatives, and regulate their cognitive effort. This second direction is adopted in the present study as the main theoretical hypothesis: critical thinking contributes to metacognition.

This choice does not imply that possible reciprocal relations should be ruled out in longitudinal studies. Rather, it allows an initial causal structure to be established for the analysis. If critical thinking is understood as a competence oriented toward explaining, deciding, and solving problems, its application may trigger processes of monitoring, planning, regulation, and evaluation that strengthen metacognition. This relation has already received empirical support in a previous study using structural equation modeling (SEM), in which critical thinking was positively associated with metacognition, which, in turn, was related to psychological well-being and empathy (Guamanga et al., 2025).

### 2.3. Academic Performance, Empathy, and Psychological Well-Being

Promoting critical thinking would make little sense if it were not oriented toward demonstrable outcomes. Incorporating socioemotional variables into this model responds precisely to this broader understanding of critical thinking. If decision-making and problem-solving require more than the proper handling of information, it is necessary to examine variables that reflect dimensions of student functioning that are not strictly cognitive. Empathy and psychological well-being make it possible to address this dimension: the former refers to understanding others and regulating social interaction, whereas the latter expresses how students relate to themselves, their goals, and the demands of their environment. From this perspective, these variables are meaningful indicators for evaluating educational impact.

Taken together, academic performance, empathy, and psychological well-being represent different but complementary criteria for evaluating student development. Academic performance reflects institutional achievement and mastery of curricular demands; empathy captures a socioemotional dimension involved in interaction, communication, and perspective taking; and psychological well-being refers to a broader form of adaptive functioning that cannot be reduced to grades or immediate satisfaction. For this reason, the present study treats these variables as distinct outcomes within the same educational system rather than as interchangeable indicators of student success.

Empathy involves adopting another person’s perspective, understanding others’ mental states, and regulating social responses, and it is closely related to reflective and metacognitive processes (Baron-Cohen, 2022; Baron-Cohen & Wheelwright, 2004; Lawrence et al., 2004; Martingano & Konrath, 2022). It may also contribute to academic performance in contexts in which interaction, communication, and cooperation are important for learning (Gilar-Corbi et al., 2020; Järvelä et al., 2018). For this reason, the present study retains empathy as a socioemotional node in the model. However, its role is interpreted as secondary in comparison with the critical thinking–metacognition–well-being axis.

From Ryff’s eudaimonic perspective, well-being is not reducible to pleasure or immediate satisfaction; rather, it expresses broader psychological functioning, organized around dimensions such as self-acceptance, environmental mastery, purpose in life, personal growth, autonomy, and positive relations with others (Ryff, 1989, 2014; Ryff & Keyes, 1995). These dimensions may be related to students’ ability to understand their experiences, adapt their strategies, overcome difficulties, and orient their actions toward meaningful goals. For this reason, metacognition may, in theory, play a fundamental role in psychological well-being (Efklides, 2006; Guamanga et al., 2025).

From this perspective, metacognition may be understood not only as a cognitive support for task performance but also as a self-regulatory resource with potential implications for students’ psychological functioning. The ability to monitor one’s own reasoning, evaluate the adequacy of strategies, and adjust behavior in response to difficulties may contribute to a greater sense of control over academic demands and to a more organized interpretation of one’s learning experience. This connection is especially relevant in higher education, where students are required to regulate effort over time, make decisions under uncertainty, and integrate academic goals with broader personal and developmental aims.

On the other hand, academic performance constitutes an institutional indicator of student achievement and makes it possible to evaluate—although only partially—the effectiveness of educational processes (Hattie, 2010; Navarro, 2003; Rivas & Saiz, 2023). In this study, performance is included as a meaningful variable because it allows us to examine whether the critical thinking–metacognition sequence is reflected in observable academic outcomes. However, academic performance does not exhaust the aims of higher education and cannot be regarded, without supporting evidence, as the main mediator between cognitive processes and well-being (Dimitrijević et al., 2018; Guamanga et al., 2024; Ryff, 2014).

### 2.4. From Previous SEM Evidence to Graphical Causal Modeling

Using structural equation modeling (SEM), a previous study analyzed the relations among critical thinking, metacognition, empathy, and psychological well-being (Guamanga et al., 2025). That work made it possible to develop a precise conceptual analysis and to empirically test a model in which critical thinking was related to metacognition, which, in turn, was related to psychological well-being and empathy. However, that model did not take academic performance into account, nor did it explicitly evaluate the conditional independence implications derived from a causal structure.

The present reanalysis is therefore not a repetition of the previous SEM study, but a reformulation of the explanatory question. Whereas the previous model estimated associative relations among psychological constructs, the present study asks whether the assumed causal structure itself is compatible with the pattern of conditional independencies observed in the data. This shift is important because two models may show plausible associations while implying different structural restrictions. By incorporating academic performance and testing a performance-mediated model against a direct metacognitive model, the present study evaluates a theoretically meaningful alternative that was not examined in the original analysis.

In this context, the present study adopts a graphical causal approach based on directed acyclic graphs (DAGs) and structural causal models (Kline, 2023; Kunicki et al., 2023; Pearl, 2009; Pearl et al., 2016; Textor et al., 2016). DAGs represent variables as nodes and hypothetical causal relations as directed arrows. This representation requires explicitly specifying which relations are assumed, which are excluded, and which conditional independencies should be observed if the theoretical structure is compatible with the data. The graphical causal approach does not replace theory or psychometric evidence; rather, it allows the structural assumptions of the model to be formulated more transparently and their testable implications to be examined.

For readers less familiar with graphical causal modeling, the central point is that a DAG does not merely display relations among variables. It also encodes assumptions about which variables should become independent once other variables are taken into account. These conditional independence implications make the model empirically testable: if the independencies implied by the graph are not observed in the data, the proposed structure requires revision. In this sense, graphical causal modeling provides a transparent way to examine whether a theoretically proposed psychological mechanism is structurally compatible with the empirical covariance pattern.

The use of a DAG is useful when the aim is to compare alternative models for the organization of a psychological system. Unlike a purely associative interpretation, graphical causal modeling requires researchers to justify the direction of the relations, identify possible mediating pathways, and evaluate whether the independencies implied by the structure correspond to the matrix of empirical relations. Therefore, the specific contribution of the present study is to extend previous SEM evidence by including academic performance and examining two possibilities: whether psychological well-being is explained primarily through a pathway mediated by academic performance or whether it is directly related to metacognition.

### 2.5. Current Study, Theoretical Model, and Structural Expectations

The present study analyzes the structural relations among critical thinking, metacognition, empathy, academic performance, and psychological well-being in university students. The initial structure proposes that critical thinking acts as an antecedent of metacognition; that metacognition is related to empathy and academic performance; that empathy contributes to performance; and that academic performance is associated with psychological well-being. This specification reflects a general mediational hypothesis: the cognitive and self-regulatory processes linked to critical thinking could translate into better academic outcomes, which, in turn, could be associated with higher levels of psychological well-being.

**Figure 2:**
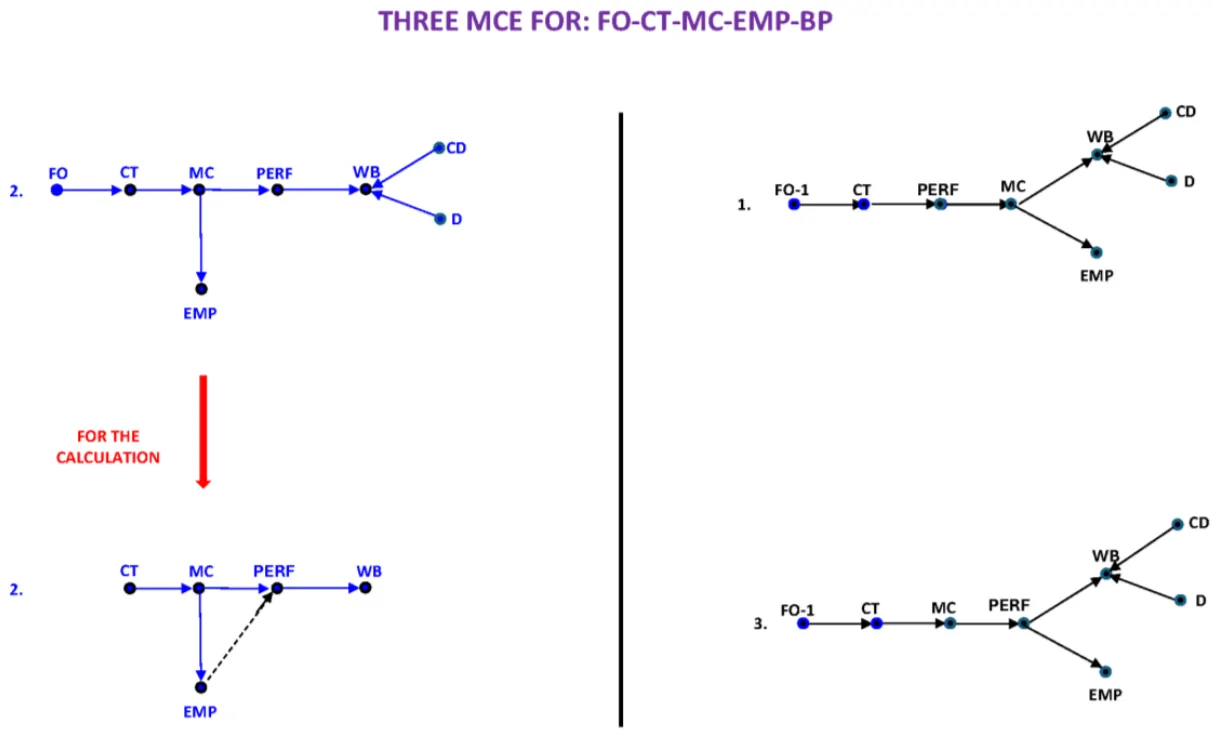
Broad theoretical causal system and empirical submodel evaluated in the present study. *Note*. CT = critical thinking; MC = metacognition; EMP = empathy; PERF = academic performance; WB = psychological well-being; FO = formative process; CD = contextual demands and changes; and D = diachronic dimension or life stage. The figure represents the broad theoretical causal system. The present study estimates only the submodel delimited by CT, MC, EMP, PERF, and WB; FO, CD, and D are retained as non-estimated conceptual nodes.

Figure 2 presents the broad theoretical causal system from which the empirical model of this study is derived (for greater clarity, see Saiz & Rivas, 2023, specifically, Fig. 4, p. 18 in the cited article). The system includes formative, cognitive, socioemotional, contextual, and diachronic factors that enable representation of the relations among education, critical thinking, performance, and well-being. However, the current analysis is restricted to the submodel for which observed data are available: critical thinking, metacognition, empathy, academic performance, and psychological well-being. This delimitation preserves the connection with the broader theoretical framework without introducing unmeasured nodes into the empirical estimation. Within this framework, the main interest is to evaluate whether the proposed causal structure generates conditional independence implications that are compatible with the data and whether the estimated paths show coherence and stability within the system.

First, critical thinking was expected to occupy an antecedent position within the model, influencing metacognition. This expectation derives from the conception of critical thinking as a competence oriented toward explanation, decision-making, and problem-solving. From this perspective, the exercise of critical thinking should activate processes of awareness, planning, monitoring, and regulation of one’s own reasoning.

Second, metacognition was specified as a mechanism linking critical thinking to academic and socioemotional outcomes. In the initial theoretical model, this function was represented through two paths: one toward academic performance and another toward empathy. The first expresses the possibility that cognitive self-regulation may translate into academic achievement; the second, that processes of monitoring and regulating one’s own thinking may be related to the understanding of others’ mental states and perspectives.

Third, empathy was proposed to contribute to academic performance. This path was included because university performance does not depend exclusively on individual cognitive skills, but also on socioemotional competences involved in interaction, communication, and academic cooperation.

Fourth, the initial model assumed a pathway to psychological well-being mediated by academic performance. According to this structure, metacognition would contribute to well-being insofar as it promotes better academic outcomes. Thus, academic performance was specified as a possible intermediate mechanism between self-regulatory processes and psychological well-being.

Finally, an alternative explanation was considered. According to this second possibility, metacognition could be directly related to psychological well-being, without this relation depending primarily on academic performance. This alternative is theoretically plausible if it is assumed that awareness, planning, and regulation of one’s own thinking support academic performance, but above all shape the way students cope with demands, interpret their experiences, and orient their actions toward meaningful goals that are not directly linked to academic requirements.

The present study represents a significant departure from previous findings by moving from an associative framework to a formal Graphical Causal Model (GCM). While earlier analyses demonstrated that Critical Thinking and Metacognition co-vary with Well-being, the causal architecture of these relationships remained a ‘black box’. By applying Directed Acyclic Graphs (DAGs) and Gaussian Bayesian Networks (GBNs), this research aims to identify the specific conditional independence constraints that govern the system, providing a more rigorous validation of the ‘metacognitive pathway’ hypothesis.

## 3. Materials and Methods

### 3.1. Data Source and Secondary Analysis Design

The present study is a secondary analysis of data from a previous line of research on critical thinking, metacognition, empathy, and psychological well-being in university students (Guamanga et al., 2025). The present work reanalyzes the dataset with a different objective: to evaluate the compatibility of a graphical causal structure that incorporates academic performance as an additional node, to examine whether the relation between metacognition and psychological well-being is organized primarily through academic performance or whether it is more compatible with a direct pathway.

Therefore, this study does not constitute a new evaluation of the effectiveness of the formative program, nor is it a replication of the previously published SEM model. Its specific contribution lies in analytically extending the available dataset through directed acyclic graphs, conditional independence tests, Gaussian Bayesian networks, and structural stability analysis (Kline, 2023; Pearl, 2009; Pearl et al., 2016; Scutari, 2010; Textor et al., 2016).

Because the present article is based on a secondary analysis, no new participants were recruited and no additional data were collected. The dataset used for the reanalysis was anonymized before the present study was conducted, and the analyses were restricted to the variables already available from the previous research project. Accordingly, the present study did not involve new contact with participants, new intervention procedures, or access to identifiable personal information. This is the basis for considering that no additional ethical clearance was required for the present secondary analysis.

### 3.2. Participants

The sample consisted of 155 psychology students from a university located in northeastern Spain. The mean age was 19.0 years (*SD* = 2.20). The sex distribution was predominantly female: 88.9% women and 11.1% men. Participants were enrolled in either the first or the final year of the undergraduate psychology program.

### 3.3. Measures and Variables

Critical thinking was assessed using the PENCRISAL test, which evaluates deductive reasoning, inductive reasoning, practical reasoning, decision making, and problem solving (Rivas & Saiz, 2012). Metacognition was measured using the Metacognitive Awareness Inventory, which assesses knowledge and regulation of cognition (Huertas Bustos et al., 2014; Schraw & Dennison, 1994). Empathy was assessed using the Empathy Quotient, an instrument designed to measure individual differences in empathy (Baron-Cohen & Wheelwright, 2004; Redondo & Herrero-Fernández, 2018). Psychological well-being was assessed using the Spanish adaptation of Ryff’s Psychological Well-Being Scale (Díaz et al., 2006; Ryff, 1989; Ryff & Keyes, 1995); for this study, the dimensions of self-acceptance, purpose in life, and environmental mastery were considered. Academic performance was operationalized through the final grade available in the dataset.

A detailed description of the sample, instruments, formative context, and data collection procedure can be found in the original study (Guamanga et al., 2025). In the present article, these measures were used as observed variables for the estimation of the graphical causal model.

### 3.4. Data Analysis

#### 3.4.1. Structural Causal Modeling (SCM) and DAG Specification

**Figure 3:**
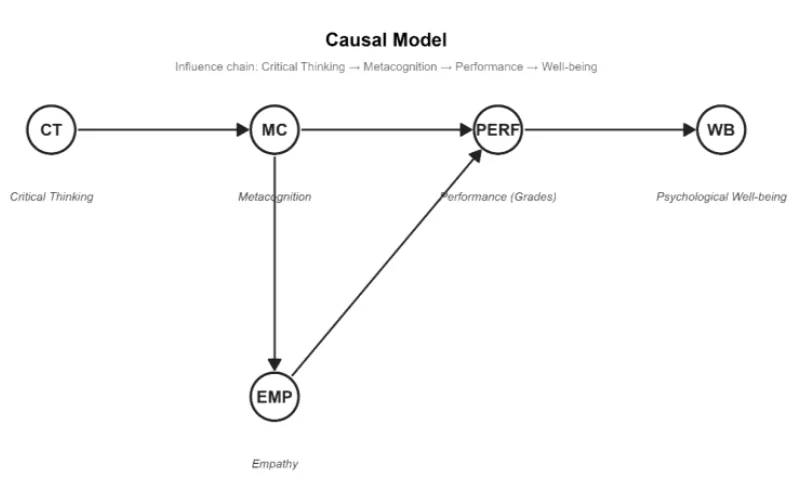
Theoretical DAG for the Performance-Mediated Hypothesis. *Note*. CT = critical thinking; MC = metacognition; EMP = empathy; PERF = academic performance; and WB = psychological well-being.

The theoretical model was formalized as a directed acyclic graph (DAG) using the dagitty R package-version 0.3-4 (Textor et al., 2016). The initial DAG represented the performance-mediated hypothesis: critical thinking (CT) was specified as an antecedent of metacognition (MC); metacognition was specified as a predictor of empathy (EMP) and academic performance (PERF); empathy was specified as a predictor of performance; and performance was specified as a predictor of psychological well-being (WB). By explicitly defining nodes and directed edges, the DAG made the structural assumptions of the model transparent and allowed us to derive the conditional independence implications required for subsequent structural diagnostics (see Figure 3).

#### 3.4.2. Identification and Structural Diagnostics

Before parameter estimation, we performed a causal identification analysis based on the backdoor criterion (Pearl, 2009). This step assessed whether the target relations were identifiable under the assumptions encoded in the DAG and conditional on the variables available in the dataset. It was not interpreted as eliminating all possible sources of confounding, but as evaluating identification within the specified graphical structure.

A critical phase of our methodology involved the evaluation of testable implications through conditional independence tests. We adopted a refinement-based diagnostic protocol: the initial theoretical model was first subjected to local independence tests to verify whether its implied d-separation statements were supported by the empirical covariance structure of the data. These structural constraints were evaluated using partial correlations as the conditional independence criterion, allowing us to examine whether the DAG’s topology was compatible with the observed linear dependencies. If any structural misfit was identified, defined as a significant violation of independence (*p* < .05), the model’s topology was systematically re-evaluated to incorporate theoretically plausible paths suggested by the diagnostics. This iterative process ensured that the final estimated model remained theoretically grounded and structurally compatible with the empirical evidence, providing the basis for subsequent parameter estimation and causal interpretation under the assumptions of the specified DAG. Consistent with the principles of graphical causal modeling (Pearl, 2009; Shipley, 2016), conditional independence tests were interpreted as global structural diagnostics rather than independent discovery tests. Consequently, no multiplicity corrections (e.g., Bonferroni) were applied, as the objective was to maintain the sensitivity of the diagnostic protocol to identify any local violation of the d-separation statements implied by the DAG.

#### 3.4.3. Gaussian Bayesian Network Parameterization

To assess the mathematical integrity of the structural model, the network was formally verified for directedness and acyclicity using the bnlearn diagnostic suite (Scutari, 2010). The final model (Model B) was confirmed as a valid directed acyclic graph (DAG), satisfying the structural prerequisites for consistent joint probability estimation and model-based counterfactual simulation.

The selected graphical structure was analyzed using Gaussian Bayesian Networks (GBN), a probabilistic framework that allows for the simultaneous estimation of local distributions for each node in the DAG. Following the implementation protocols for GBNs (Scutari, 2010), we employed Maximum Likelihood Estimation (MLE) to fit the network parameters. Under this framework, each node is modeled as a local linear regression, where the relevant parameters, regression coefficients for each parent and the standard deviation of the residuals, are estimated to be consistent with the global network topology. The statistical adequacy of this approach was verified through residual diagnostics; univariate normality was confirmed for all local distributions, with absolute values of skewness (range: 0.00 to 0.82) and kurtosis (range: 0.19 to 1.46) well within the recommended thresholds of 2.0 and 7.0, respectively (Kline, 2023).

Unlike independent regression models, which estimate path coefficients in isolation, the GBN approach estimates the joint probability distribution of the system. As established by Pearl (2009), this systemic estimation is relevant for the structural interpretation of the model, because it allows the local dependencies to be represented within the global graphical architecture. To ensure statistical replicability and facilitate the comparison of effect sizes, all variables were standardized (Z-scores). We extracted standardized coefficients (*β*), standard errors, and *p*-values for each local node using the bnlearn (version 5.1) and stats packages in R version 4.5.2 (R Core Team, 2026), providing a granular yet systemically consistent view of the specified graphical structure.

Finally, the structural stability of the identified arcs was evaluated using non-parametric bootstrapping (R = 100,000 replicates). This procedure calculates the “arc strength,” the empirical probability of each connection appearing across multiple resampled datasets. This diagnostic step was used to assess the robustness of the selected graphical structure to sampling variation, particularly given the specific study sample (N = 155), and to provide additional evidence for the stability of the estimated adjacencies.

The standardized regression coefficients (β) reported in this study are directly comparable in magnitude and interpretation to the path coefficients typically found in Structural Equation Modeling (SEM). This allows for a consistent comparison with previous associative findings (Guamanga et al., 2025), clarifying that the GBN estimates represent the same units of standardized change within a probabilistic graphical framework.

#### 3.4.4. Predictive Validity and Causal Inference Simulation

To assess the practical utility and robustness of the model, we employed an analytic approach combining predictive performance and model-based counterfactual simulation. First, the predictive adequacy of the graphical structure was examined through local node diagnostics and goodness-of-fit metrics. We calculated the Adjusted R-squared ($R_{adj}^{2}$) and the Root Mean Square Error (RMSE) for the primary outcome, psychological well-being (WB). These metrics provide an empirical measure of the model’s explanatory power, indicating the proportion of variance in students’ psychological well-being accounted for by the specified predictors, while adjusting for model complexity.

Subsequently, to explore the model-implied systemic impact of hypothetical changes in critical thinking, we performed a large-scale Monte Carlo simulation (n = 1,000,000) based on the principles of Pearl’s do-calculus (Pearl, 2009). We simulated a hypothetical structural intervention on critical thinking, denoted as do (CT), to estimate the model-implied total causal effect on psychological well-being (WB). By comparing the expected value of the outcome under two counterfactual scenarios, High CT (>0 *SD*) versus Low CT (<0 SD), we estimated how changes in CT would propagate through the specified graphical structure under the assumptions of the model.

This integrated approach complements simple path analysis by combining descriptive predictive diagnostics with an exploratory counterfactual projection under the specified DAG. By accounting for the interconnected topology of the selected model, the simulation describes the propagation of a hypothetical intervention through the modeled paths. Therefore, the resulting estimate should be interpreted as a model-based counterfactual projection, not as evidence of the observed effectiveness of an educational intervention.

## 4. Results

### 4.1. Structural Diagnostics and Model Refinement

The initial theoretical model (Model A) was subjected to local independence tests to verify its structural consistency with the empirical data. As shown in Table 1, while several d-separation statements were supported (e.g., CT⊥EMP∣MC, p=.800), the model showed a significant violation of the independence assumption between Metacognition and Well-being given Performance (MC⊥WB∣PERF, p<.001). The diagnostics revealed a strong residual association ($estimate=0.50,\;p=1.32\times 10^{-11}$), indicating that the theoretical assumption of full mediation through academic achievement was empirically unsupported.

Following the diagnostic protocol, the DAG was refined by incorporating a direct path from Metacognition to Well-being (Model B). This structural adjustment resulted in a statistically adequate model in which all testable implications were supported by the empirical evidence (all *p*-values >.05). This refined topology served as the foundation for the subsequent parameter estimation. While the transition from Model A to Model B is data-driven, it represents a structural correction of a previous theoretical omission. Consequently, Model B should be interpreted as a refined hypothesis derived from causal diagnostics, setting a new structural baseline for future confirmatory studies.

The rejection of Model A specifically highlights that the autonomy of the process in relation to psychological well-being was underestimated in previous associative models. The significant residual association uncovered by the d-separation test (estimate = 0.50, p<.001) indicates that the benefits of metacognitive competence are not strictly instrumental to academic success (Performance). Instead, metacognition appears to contribute directly to the individual’s sense of environmental mastery and purpose, independent of their formal grades. This diagnostic finding was the empirical catalyst for the structural refinement that led to Model B.

### 4.2. Parameter Estimation

The Gaussian Bayesian Network (GBN) estimation for Model B revealed that Critical Thinking (CT) showed a significant positive path to Metacognition (MC) (β=0.21, SE=0.08, p=.009). Notably, the direct path from Metacognition to Psychological Well-being emerged as the strongest estimated path in the model (β=0.50, SE=0.07, p<.001).

**Figure 4:**
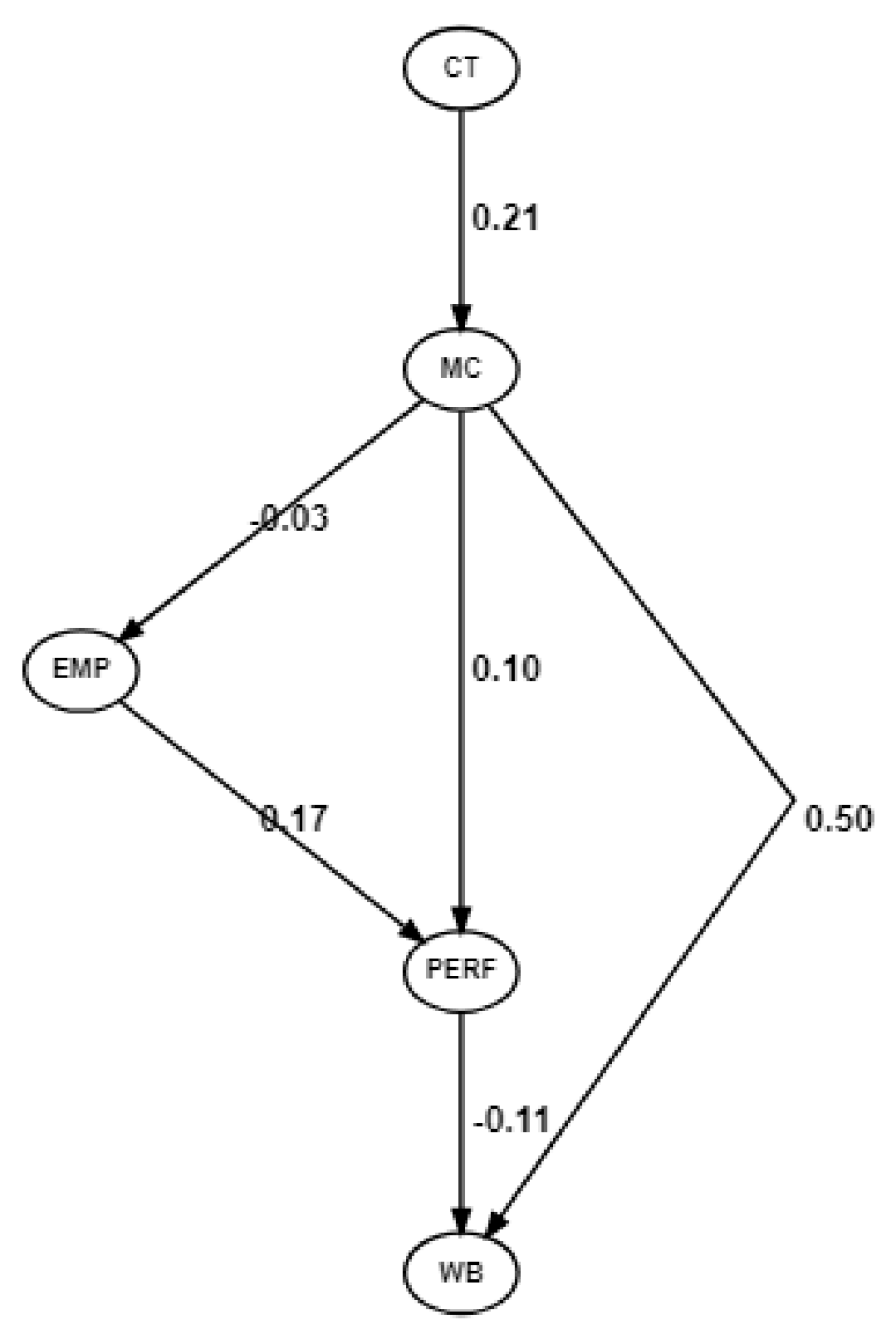
Gaussian Bayesian Network (GBN) parameters for the Refined Model (Model B). *Note*. CT = critical thinking; MC = metacognition; EMP = empathy; PERF = academic performance; and WB = psychological well-being.

Regarding academic achievement, Empathy was a significant predictor of Performance (β=0.17, SE=0.08, p=.036), while the direct effect of Metacognition on grades was non-significant (β=0.10, SE=0.08, p=.219). Furthermore, the association between Performance and Well-being was weak and non-significant (β=−0.11, SE=0.07, p=.115), suggesting that psychological well-being in this sample was more closely associated with metacognitive self-regulation than with academic success (see Figure 4).

### 4.3. Structural Stability Analysis

To assess the empirical stability of the adjacencies within this sample, an extensive non-parametric bootstrap analysis was performed (R = 100,000 replicates). This procedure estimates the “Arc Strength” (Friedman et al., 1999), which represents the empirical probability of a connection appearing across resampled datasets. While bootstrap resampling does not substitute for large-sample power, it provides a rigorous measure of structural confidence within the current sample. As shown in Table 2, the results confirmed the high structural stability of the core adjacencies.

Most notably, the adjacency between Metacognition and Well-being demonstrated very high arc strength (strength = 0.9999), appearing in almost all simulations. The cognitive axis from Critical Thinking to Metacognition also showed solid structural support (strength = 0.618). In contrast, paths involving academic performance exhibited lower stability (range: 0.16 to 0.44), reflecting the multifactorial and volatile nature of student grades.

These values should be interpreted as indicators of high internal consistency of the model given the current dataset (N = 155). Since the MC→WB path was incorporated following the diagnostic failure of Model A, the high arc strength (0.999) corroborates that this dependency is a persistent and stable feature of the data-generating process in this specific sample, rather than an artifact of localized noise. However, this internal stability does not constitute cross-validation, and its generalizability remains to be tested in external populations.

### 4.4. Causal Inference Simulation

The predictive adequacy of the selected model was evaluated through local node diagnostics for the primary outcome. The model demonstrated an Adjusted $R^{2}$ of 0.244 for Psychological Well-being (WB), indicating that the specified paths, including the direct metacognitive path and the indirect effects propagated from Critical Thinking and Empathy, account for 24.4% of the total variance in students’ psychological well-being. This represents a moderate-to-large effect size for behavioral data, according to the benchmarks established by Cohen (1988), where $R^{2}\approx 0.26$ is considered a large effect in social sciences.

Subsequently, a Monte Carlo simulation (n=1,000,000) was employed to estimate the total causal effect of an intervention on Critical Thinking (do(CT)) over Psychological Well-being (WB). The simulation yielded a Total Causal Effect of 0.164 (95% CI [0.13, 0.20]). This result indicates that a one-standard-deviation increase in critical thinking was associated, under the assumptions of the specified DAG, with an expected increase of 0.164 standard deviations in well-being. This estimate should be interpreted as a model-based counterfactual projection, not as evidence of the observed effectiveness of an educational intervention, and it remains subject to the structural assumptions encoded in Model B.

## 5. Discussion

The main finding of the study was that the relation between metacognition and psychological well-being was not adequately explained by academic performance. The conditional independence tests rejected the complete mediation proposed in the initial DAG and favored a refined model with a direct path between metacognition and psychological well-being.

The limited role of academic performance constitutes one of the most informative results of the model. Although the hypothesis of a performance-mediated pathway was theoretically plausible, the metacognition → performance and performance → well-being paths did not receive statistical support. Academic performance retains value as an educational outcome, but it did not operate as the main link between metacognition and psychological well-being in this sample. This interpretation is consistent with the literature that understands grades as partial indicators of academic functioning, dependent on cognitive, motivational, institutional, and contextual conditions (Gilar-Corbi et al., 2020; Mammadov, 2022; Richardson et al., 2012).

The direct relation between metacognition and psychological well-being is central to the interpretation of the model. Metacognition was not associated only with academic functioning, but also with the way students may monitor, regulate, and organize their own experience. This reading is consistent with classical definitions of metacognition as knowledge, monitoring, and regulation of cognitive processes (Flavell, 1979; Schraw & Dennison, 1994; Schraw & Moshman, 1995), as well as with models of self-regulated learning that emphasize planning, strategic control, and evaluation of one’s own performance (Pintrich, 2000; Zimmerman, 2002). It is also consistent with approaches that link metacognition to affective experience and the regulation of one’s own cognitive activity, as well as with previous evidence on its relation to psychological well-being (Efklides, 2006; Guamanga et al., 2025). From this perspective, psychological well-being appears to be more closely linked to processes of metacognitive monitoring and regulation than to grades alone.

At a comparative level, our re-analysis reveals a nuance that was previously obscured: the ‘Academic Performance’ node, often centralized in educational studies, showed remarkably lower structural stability (volatility) compared to the ‘Metacognition → Well-being’ axis. Our observation suggests that while grades are subject to multifactorial noise and external institutional variables, the link between thinking about one’s thinking and feeling psychologically well is a highly stable internal mechanism. This insight, made possible only by the structural stability analysis (bootstrap), shifts the focus from an achievement-centered model to a competence-centered model of student development. Specifically, while earlier analyses of this dataset emphasized cumulative associations, our graphical approach identifies the ‘metacognitive pathway’ as the most resilient structural feature of the system, surviving 99.9% of the resampling simulations.

A critical methodological consideration in our GBN model is the directionality of the MC→WB  path. From a purely mathematical perspective, the model is Markov-equivalent to WB→MC, as both would imply the same conditional dependencies observed in the data. However, our choice of MC→WB is rooted in the theoretical framework of metacognitive self-regulation, which posits cognitive monitoring as a functional antecedent to psychological adjustment. While our data confirms the existence of a high-stability structural link between these nodes, we acknowledge that in a cross-sectional design, the causal direction remains a theoretical postulate that should be further tested using longitudinal or experimental data.

The remaining paths help situate the roles of critical thinking and empathy within the system. The positive path between critical thinking and metacognition is consistent with a conception of critical thinking oriented toward explanation, decision making, and problem solving: thinking critically requires examining evidence, comparing alternatives, anticipating consequences, and revising one’s own judgment. This interpretation is consistent with approaches that define critical thinking as self-regulated judgment, analysis, inference, evaluation, and reasoned decision making (Facione, 1990; Halpern & Dunn, 2023), and with proposals that place causal explanation and problem solving at the center of critical thinking instruction (Guamanga et al., 2023; Rivas et al., 2023; Saiz, 2020). Empathy, by contrast, showed a more limited role, mainly associated with academic performance, suggesting a socio-academic contribution rather than a central position in explaining well-being (Baron-Cohen & Wheelwright, 2004; Davis, 1983; Gilar-Corbi et al., 2020).

The findings should be interpreted as evidence of structural compatibility, not as definitive proof of causal effects. The DAG made it possible to formalize theoretical assumptions, evaluate conditional independence implications, and compare two explanatory structures. However, the strength of the causal interpretation depends on the plausibility of the specified graph, the variables measured, and the absence of relevant unobserved confounders (Kline, 2023; Pearl, 2009; Textor et al., 2016). Within these limits, the model supports a more precise interpretation: critical thinking is linked to psychological well-being mainly through metacognitive processes, without academic performance functioning as the main explanatory mechanism.

This finding is particularly relevant because it specifies what the reanalysis adds to the previous evidence. The value of the present model does not lie in obtaining stronger associations among the same variables, but in testing whether a theoretically plausible mediation through academic performance is structurally compatible with the data. The results suggest that this was not the case. Therefore, the contribution of the reanalysis is to delimit the explanatory role of academic performance and to identify metacognitive self-regulation as the more coherent pathway linking critical thinking and psychological well-being within the specified graphical structure.

The most specific contribution of the study lies in the relation between metacognition and psychological well-being. From Ryff’s eudaimonic model, well-being involves dimensions such as self-acceptance, purpose in life, environmental mastery, autonomy, personal growth, and positive relations with others (Ryff, 1989, 2014; Ryff & Keyes, 1995). These dimensions do not depend solely on external outcomes, such as grades, but on how individuals interpret their experiences, regulate their actions, and sustain meaningful goals. The metacognition → psychological well-being path is consistent with this perspective: students with greater monitoring and regulation capacity may cope more effectively with academic demands and organize their formative experience with greater clarity (Efklides, 2006; Guamanga et al., 2025; Schraw & Dennison, 1994).

The secondary role of academic performance helps qualify previous studies on critical thinking, well-being, and achievement. Guamanga et al. (2024) found that critical thinking and psychological well-being contributed to academic performance, with critical thinking contributing more strongly. The present study examines a different question: whether performance explains the relation between metacognition and well-being. The results did not support this complete mediation. This difference does not contradict the previous study; rather, it clarifies the place of performance within the system. Grades may be an important outcome of education, but they did not exhaust the explanation of psychological well-being in the estimated model. This interpretation is consistent with the literature that characterizes academic performance as a multidetermined phenomenon, influenced by cognitive abilities, motivation, self-regulation, prior achievement, personality, and contextual conditions (Gilar-Corbi et al., 2020; Mammadov, 2022; Richardson et al., 2012; Robbins et al., 2004).

Empathy occupied a more delimited position within the model. Its association with academic performance suggests a socioacademic function linked to interaction, communication, and understanding others in learning contexts. However, empathy did not organize the relation among critical thinking, metacognition, and psychological well-being. This pattern is compatible with a differentiated reading of the system: metacognition articulates the self-regulatory dimension of the model, whereas empathy appears to contribute more specifically to academic and social functioning.

The methodological contribution of the study lies in articulating psychological theory with graphical causal testing. Pearl (2009) and Pearl and Mackenzie (2018) have shown that causal models require the explicit specification of assumed relations, included variables, and independencies derived from the graph. Textor et al. (2016) developed this logic in the applied use of DAGs for causal inference. In psychology and education, this strategy can complement the use of SEM to evaluate complex theoretical models (Kline, 2023), because it requires researchers to examine whether the model’s structural restrictions are compatible with the data. In this study, the combination of a DAG, Gaussian Bayesian networks, and conditional independence tests made it possible to compare two explanations: a performance-mediated pathway and a direct self-regulatory pathway. The latter offered a more coherent representation of the system analyzed.

The results suggest that university instruction in critical thinking should explicitly incorporate metacognitive self-regulation tasks. If the structure most compatible with the data places metacognition as the main link between critical thinking and psychological well-being, teaching should not be limited to producing better arguments or better grades. Formative activities should require students to explain how they reasoned, which assumptions they used, which evidence they considered, which alternatives they rejected, and how they would evaluate the quality of their decision (Halpern & Dunn, 2023; Kuhn & Dean, 2004; Pintrich, 2000; Zimmerman, 2002). Thus, this type of education is framed less around theoretical content than around the exploration of students’ experience and, especially, around the practical “doing” component that places them before real problems, even when these processes are not directly reflected in grades.

This orientation is consistent with an applied conception of critical thinking. Thinking critically involves explaining problems, identifying causes, comparing alternatives, making reasoned decisions, and evaluating the consequences of action. Therefore, the sequence explain–decide–solve–produce change should be translated into observable instructional procedures: case analysis, formulation of causal hypotheses, evaluation of evidence, review of alternatives, decision making, and subsequent evaluation of the results obtained (Saiz, 2020; Saiz & Rivas, 2023; Guamanga et al., 2023). Within this framework, metacognition makes it possible to transform task resolution into conscious, revisable, and transferable learning.

The results also suggest that grades should not be the only criterion for evaluating a critical thinking intervention. Academic performance retains value as an educational indicator, but in this study, it did not operate as the main link between metacognition and psychological well-being. For this reason, alongside performance indicators, it would be advisable to include measures of metacognitive monitoring, explanatory quality, error revision, decision justification, transfer to new contexts, and perceived control over one’s own learning. The association between empathy and academic performance adds a complementary implication: tasks involving discussion, explanation, and problem solving may incorporate interaction, listening, and perspective taking when learning requires cooperation.

In the context of generative artificial intelligence, the educational implication is concrete: students must learn to retain control over their reasoning. AI can provide decisive support in the search, synthesis, and reorganization of information. However, its critical evaluation—that is, reasoned judgment about the quality of a response, the reconstruction of an explanation, the detection of biases, and the final decision—remains an exclusively human responsibility. Therefore, fostering critical thinking in this context requires teaching students to formulate good questions, contrast sources, review automated explanations, and justify when a solution is acceptable. In other words, because these reflective processes directly affect individuals’ decisions and well-being, critical thinking instruction also involves non-cognitive aspects, such as epistemic integrity and the intellectual responsibility to act in accordance with what one believes or knows (Ennis, 1996; Facione, 1990; Hamby, 2015).

These implications should be interpreted with caution. The study does not demonstrate that an intervention in critical thinking produces direct improvements in psychological well-being. Rather, it shows that, under the specified graphical causal structure, metacognition occupies an important position in the relation between critical thinking and well-being. The next step is to evaluate whether programs aimed at jointly strengthening critical thinking and metacognition produce observable changes in self-regulation and, subsequently, in psychological well-being.

The main limitation of the study concerns the scope of causal inference. The graphical causal approach made it possible to specify theoretical assumptions, derive conditional independence implications, and compare alternative structures. However, the empirical design does not allow experimental causality to be established. The estimated relations should be interpreted as compatible with the specified DAG and its assumptions, not as conclusive evidence of causal effects. DAGs formalize causal hypotheses, but they do not eliminate the possibility of residual confounding, omitted variables, or plausible alternative directions (Kline, 2023; Pearl, 2009; Textor et al., 2016).

The second limitation concerns the temporality of the data. The model proposes a sequence among critical thinking, metacognition, academic performance, empathy, and psychological well-being, but these processes may develop dynamically and reciprocally. Metacognition may promote well-being, although well-being may also influence students’ disposition to plan, monitor, and sustain cognitive effort. Future research should use longitudinal designs to evaluate intraindividual change and more rigorously test the temporal direction of the relations (Kline, 2023).

The empirical model analyzed represents only part of the broader theoretical system. The conceptual framework includes formative, contextual, and diachronic factors that were not measured in this dataset. Therefore, the estimated submodel should not be interpreted as a complete representation of the link among education, critical thinking, and well-being. Subsequent studies should incorporate variables such as intervention characteristics, academic demands, prior achievement, motivation, social support, academic satisfaction, and stage in the formative cycle.

The size and composition of the sample (N=155) limit the generalizability of the results. Although Gaussian Bayesian Networks (GBN) are computationally efficient and can yield stable Maximum Likelihood Estimates in sparse graphs with moderate samples, we acknowledge that the high ‘Arc Strength’ observed in our bootstrap analysis reflects empirical stability within this specific dataset rather than universal external validity. Replication is needed in larger, more heterogeneous samples drawn from different programs and institutions. Such replication is especially necessary for the paths linked to academic performance and empathy, which showed lower stability than the core relation between metacognition and psychological well-being.

Finally, the counterfactual simulation should be interpreted as an exploratory tool (Pearl & Mackenzie, 2018). Its value lies in estimating how hypothetical changes in critical thinking could propagate within the specified structure, but it does not replace evidence from an actual intervention. The next empirical step is to evaluate programs aimed at jointly strengthening critical thinking and metacognition through longitudinal, quasi-experimental, or experimental designs. Only with this type of evidence will it be possible to determine whether critical thinking instruction strengthens metacognition and whether these changes subsequently contribute to the psychological well-being of university students.

## 6. Conclusions

The present study showed that the structure most compatible with the data was the direct pathway between metacognition and psychological well-being. The model initially mediated by academic performance did not sufficiently describe this relation, whereas the refined model positioned metacognition as the main node for interpreting the link between critical thinking and well-being.

The substantive contribution of the study lies in clarifying the role of academic performance within the system. Grades retained value as an educational outcome, but they did not function as the main mechanism between metacognition and psychological well-being. Empathy, in turn, showed a more delimited relation with academic performance, compatible with a socioacademic function in contexts of interaction and cooperation.

The methodological contribution lies in the use of a DAG and Gaussian Bayesian networks to compare alternative explanatory structures. These procedures made it possible to formalize assumptions, evaluate conditional independence implications, and estimate effects under the specified model. The conclusions, however, should be interpreted as evidence of structural compatibility, not as experimental proof of causality. The next step will be to evaluate, through longitudinal or intervention-based designs, whether critical thinking instruction strengthens metacognition and whether these changes subsequently contribute to psychological well-being.

In the current context of higher education, these findings are relevant because they shift the interpretation of critical thinking from a narrowly academic outcome toward a broader self-regulatory and developmental process. The study suggests that the educational value of critical thinking should not be assessed only through grades, but also through its relation to metacognitive monitoring, regulation of learning, and psychological well-being. This is especially important in contemporary learning environments, where students must evaluate information, make decisions, and preserve responsibility for their reasoning in contexts increasingly mediated by artificial intelligence. Thus, the present reanalysis contributes to the field by clarifying the specific place of academic performance within the model and by highlighting metacognition as a central mechanism for connecting critical thinking with student well-being.

## Figures and Tables

**Table 1:** Structural Diagnostic Tests (Local Independence).

Implied Independence (d-Separation)	Model A (Theoretical)	Model B (Refined)	Result/Interpretation
CT⊥EMP∣MC	p=.800	p=.800	Supported
CT⊥PERF∣MC	p=.771	p=.771	Supported
CT⊥WB∣MC	p=.925	p=.925	Supported
CT⊥WB∣PERF	p=.227	--	--
EMP⊥WB∣PERF	p=.294	--	--
EMP⊥WB∣{MC,PERF}	--	p=.371	Supported
MC⊥WB∣PERF	p<.001	--	Rejected in Model A

*Note*: p<.05 indicates a violation of structural assumptions.

**Table 2:** Structural Stability and Arc Strength (Bootstrap R = 100,000).

Causal Path (From → To)	Arc Strength	Direction Confidence	Interpretation
CT → MC	0.618	0.504	Stable
MC → WB	0.999	0.476	Highly Stable
EMP → PERF	0.440	0.510	Moderate
MC → PERF	0.227	0.231	Volatile
PERF → WB	0.168	0.844	Volatile
MC → EMP	0.046	0.474	Weak

*Note*: Strength represents the proportion of bootstrap samples in which the arc was present.

## Data Availability

The data supporting the findings of this study are not publicly available due to privacy and ethical restrictions involving student data. The data may be made available from the corresponding author upon reasonable request and subject to institutional and ethical approval.
